# Supplementary material for: Tetrodotoxin Profiles in Xanthid Crab Atergatis floridus and Blue-Lined Octopus Hapalochlaena cf. fasciata from the Same Site in Nagasaki, Japan
Source: Toxins (Basel). 2023 Mar 3;15(3):193. doi: 10.3390/toxins15030193 (PMC10052739; doi:10.3390/toxins15030193)
Supplement: Supplementary file 1 [file toxins-15-00193-s001.zip › toxins-2241466-supplementary.pdf]

toxins

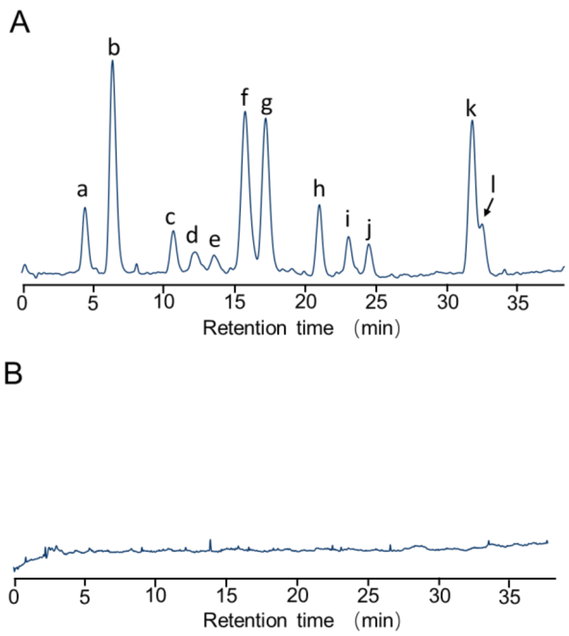

Figure S1. HPLC-FLD chromatograms of **A**: PSTs standard and **B**: *A. floridus* extracts in 2020.

a: dcneoSTX; b: dcSTX; c: dcGTX3; d: hyneoSTX; e: hySTX; f: neoSTX; g: STX; h: dcGTX2; i: GTX3; j: GTX4; k: GTX1; l: GTX2.
